# The gauge-invariant Lagrangian, the Power–Zienau–Woolley picture, and the choices of field momenta in nonrelativistic quantum electrodynamics

**DOI:** 10.1038/s41598-021-94405-z

**Published:** 2021-08-11

**Authors:** A. Vukics, G. Kónya, P. Domokos

**Affiliations:** Wigner Research Centre for Physics, P.O. Box 49., 1525 Budapest, Hungary

**Keywords:** Quantum optics, Atomic and molecular interactions with photons, Theoretical physics

**arising from**: E. Rousseau and D. Felbacq; *Scientific Reports*10.1038/s41598-017-11076-5 (2017).

## Introduction

We show that the Power–Zienau–Woolley picture of the electrodynamics of nonrelativistic neutral particles (atoms) can be derived from a gauge-invariant Lagrangian without making reference to any gauge whatsoever in the process. This equivalence is independent of choices of canonical field momentum or quantization strategies. In the process, we emphasize that in nonrelativistic (quantum) electrodynamics, the always appropriate generalized coordinate for the field is the transverse part of the vector potential, which is itself gauge invariant, and the use of which we recommend regardless of the choice of gauge, since in this way it is possible to sidestep most issues of constraints. Furthermore, we point out a freedom of choice for the conjugate momenta in the respective pictures, the conventional choices being appropriate in the sense that they reduce the set of system constraints.

Let us consider a neutral atom consisting of nonrelativistic point particles coupled to the electromagnetic field. To describe this system either classically or quantum mechanically, one can use different formulations of the same dynamics. One approach uses the minimal-coupling Hamiltonian, while another approach uses the multipolar Hamiltonian. This latter Hamiltonian is conventionally derived through a unitary transformation, which is referred to as the Power–Zienau–Woolley (PZW) transformation.

In a recent article, Rousseau and Felbacq^1^ argue that the derivation of the multipolar Hamiltonian based on the PZW transformation is inconsistent, based on consideration of gauges, constraints, and choices of canonical momenta. These claims call into question a significant body of work in nonrelativistic quantum electrodynamics, research including a recent paper of ours^2–9^, as well as textbooks^10–12^. In this paper, we clearly refute these concerns.

In particular, we show (“Power–Zienau–Woolley transformation on the Lagrangian”) that the PZW transformation can be directly performed at the level of the Lagrangian formalism, even before any Hamiltonians are constructed. Furthermore, it is possible to use a gauge-invariant form of the Lagrangian of the minimal-coupling picture as the starting point of such a derivation. This shows that the PZW transformation cannot be declared inconsistent based on arguments about gauge or canonical momentum, since the equivalence of the PZW picture with the minimal-coupling one is demonstrated in a completely gauge-invariant manner at the level of the Lagrangian, even before the canonical momentum variables and the Hamiltonian are introduced (which we will do only in “The Hamiltonian in the PZW picture”).

Our approach here is based on the use of the *transverse part of the vector potential* (${\mathbf{A }}_{\bot }$) as generalized field coordinate, which is also gauge invariant (a gauge transformation changes only the scalar potential and the longitudinal part of the vector potential). We advocate the use of this coordinate in whatever picture or gauge. This choice is superior to using the full vector potential (${\mathbf{A }}$) in some gauge not only because the gauge-invariance of the treatment would be lost, but also because with ${\mathbf{A }}$ as coordinate, the particle and field degrees of freedom would be mixed, leading to an inconvenient set of system constraints (or, nontrivial commutation relations in the quantum case). With our choice, the treatment of constraints is not an issue, and there remains only a single nontrivial commutation relation, which is moreover the same in both the minimal-coupling and PZW pictures.

In the case of constrained systems, the real power of the Lagrangian formalism is manifested when the constraints can be treated by an appropriate choice of reduced coordinates that bijectively cover the hyperplane in configuration space (c-space) defined by the constraints—this hyperplane is called configuration manifold (c-manifold). (When this is not possible, the more general method of the Lagrangian multiplier must be used.) Then, one does not need to worry about the constraints anymore, since they are encoded in the very choice of generalized coordinates. This is the case with the ideal pendulum, where we can choose the angle of the pendulum instead of the Cartesian coordinates to cover the c-manifold. This is also the case in the eletrodynamics of nonrelativistic charged particles, where we can choose ${\mathbf{A }}_{\bot }$ plus the particle coordinates to cover the c-manifold in a bijective manner. (This is not the case in the electrodynamics of relativistic particles—fields, where the covariant formulation necessitates the use of the full potential four-vector).

**Figure 1:**
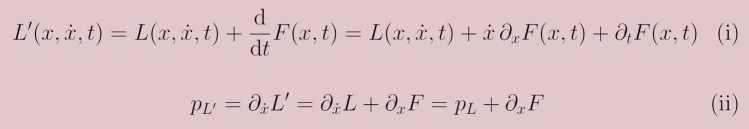
Change of conjugate momentum under an equivalent transformation of the Lagrangian $L$ defined by $F$, a function of the generalized coordinates and time.

In this paper, the PZW transformation is introduced as an equivalent transformation of the Lagrangian. Such transformations do not change the generalized coordinates because these are in fact part of the definition of the Lagrangian problem. Without saying what the generalized coordinates and velocities are, as a function(al) of which the Lagrangian is considered, the problem is ill-defined. On the other hand, the momenta conjugate to the coordinates do change under an equivalent transformation, which is the case already in point mechanics, cf. Fig. 1.

Note that in the formulation when the PZW transformation is treated as a unitary transformation in the quantum case, the transformation operator commutes with ${\mathbf{A }}_{\bot }$ so that the coordinate remains unchanged there as well.

Another important observation is that once the set of generalized coordinates is fixed, the conjugate field momenta still exhibit a certain ambiguity, which is unknown in point mechanics, being field-theoretic in nature. “Ambiguity of the field momenta” is devoted to the analysis of this phenomenon. The point is that without changing the Lagrangian (and hence without changing the gauge), due to that spatial integrals of certain combinations of fields satisfying certain relations (including gauge conditions) vanish, different functional derivatives can be extracted. If one works in the minimal coupling picture, a longitudinal vector field can be freely added to the canonical momentum. This allows one to replace the electric field with its transverse part. In the PZW picture, an analogous freedom is present: we find that here the displacement field and the transverse electric field are two legitimate choices for the canonical field momentum.

## Fundamentals

We consider a neutral atom consisting of nonrelativistic point particles and interacting with the electromagnetic field. (The reason why it is important to consider only one atom will be explained in “The connection with Poincaré gauge”.) Although the present section largely consists of textbook material, we give a succint account of these foundations that will be pertinent to the delicate questions of canonical coordinates-momenta and gauges.

### Maxwell equations

The two homogeneous Maxwell equations read:

1a $$\begin{aligned} \varvec{\nabla } \times \mathbf{E }&= - \partial _{t} {\mathbf{B }} , \end{aligned}$$

1b $$\begin{aligned} \varvec{\nabla } \cdot \mathbf{B }&= 0 , \end{aligned}$$

and the two inhomogeneous ones:

1c $$\begin{aligned} \varvec{\nabla } \times \mathbf{B }&= \mu _0 \, {\mathbf{j }} + \varepsilon _0\, \mu _0 \, \partial _{t} {\mathbf{E }} , \end{aligned}$$

1d $$\begin{aligned} \varvec{\nabla } \cdot \mathbf{E }&= \frac{1}{\varepsilon _0} \, \rho . \end{aligned}$$

The source terms of the equations are the charge and current densities, which for point-like particles read respectively:

2a $$\begin{aligned} \rho (\mathbf{x },t)&= \sum _{\alpha =0}^{Z} \, q_{\alpha } \, \delta \left( \mathbf{x }- \mathbf{x }_\alpha (t)\right) , \end{aligned}$$

2b $$\begin{aligned} {\mathbf{j }} (\mathbf{x },t)&= \sum _{\alpha =0}^{Z} \, q_{\alpha } \, {\dot{\mathbf{x }}}_\alpha (t) \, \delta \left( \mathbf{x }- \mathbf{x }_\alpha (t) \right) . \end{aligned}$$

Here $\alpha$ indexes the particles constituting the atom of atomic number $Z$, with $\alpha =0$ denoting the nucleus. From the two inhomogeneous Maxwell equations, it can be shown that they satisfy the continuity equation:

3 $$\begin{aligned} \partial _t \rho + \varvec{\nabla } \cdot \mathbf{j } = 0 , \end{aligned}$$

which is needed also for the consistency of the Maxwell equations. The particle motion is governed by the Lorentz force:

4 $$\begin{aligned} m_{\alpha } \, \ddot{\mathbf{x }}_\alpha (t) = q_{\alpha } \big [ {\mathbf{E }} (\mathbf{x }_\alpha (t),t) + {\dot{\mathbf{x }}}_\alpha (t) \times {\mathbf{B }} (\mathbf{x }_\alpha (t),t) \big ] . \end{aligned}$$

### The potentials and the Coulomb gauge

The two homogeneous Maxwell equations can be automatically satisfied by deriving the fields from potentials as

5a $$\begin{aligned} {\mathbf{E }}&= -\partial _{t} {\mathbf{A }} - \varvec{\nabla }\Phi , \end{aligned}$$

5b $$\begin{aligned} {\mathbf{B }}&= \varvec{\nabla } \times {\mathbf{A }} , \end{aligned}$$

$\Phi$ being the scalar, while ${\mathbf{A }}$ the vector potential. They can be subjected to gauge transformations that leave the physical fields unchanged:

6a $$\begin{aligned} \Phi '&= \Phi + \partial _t \chi , \end{aligned}$$

6b $$\begin{aligned} {\mathbf{A }}'&= {\mathbf{A }} - \varvec{\nabla }\chi . \end{aligned}$$

Here, $\chi$ is an arbitrary scalar field. Such transformations constitute the main theme of the discussion at hand.

The most common choice of gauge in the electrodynamics of atoms is the Coulomb gauge, defined by

7 $$\begin{aligned} \varvec{\nabla } \cdot {\mathbf{A }}_{\text {C}} =0 . \end{aligned}$$

Then, the transverse and longitudinal components of the electric field read:

8a $$\begin{aligned} {\mathbf{E }}_{\bot }&= - \partial _{t} \, {\mathbf{A }}_{\text {C}} , \end{aligned}$$

8b $$\begin{aligned} {\mathbf{E }}_{\Vert }&= - \varvec{\nabla }\Phi _{\text {C}} . \end{aligned}$$

Where by definition the transverse component is divergence-while the longitudinal is curl-free (Helmholtz decomposition). Moreover, according to the Maxwell equation (1d) we have

9 $$\begin{aligned} {\nabla }^{2}{\Phi _{\text {C}}} = -\frac{1}{\varepsilon _0} \, \rho \; \Longrightarrow \; \Phi _{\text {C}}\left( {\mathbf{x }},t\right) =\frac{1}{4\pi \varepsilon _0}\int \hbox {d}^{3}{x'}\frac{\rho \left( {\mathbf{x }}',t\right) }{| {\mathbf{x }}'-{\mathbf{x }} |}= \frac{1}{4\pi \varepsilon _0}\sum _{\alpha =0}^Z\frac{q_\alpha }{| {\mathbf{x }}_\alpha (t)-{\mathbf{x }} |}, \end{aligned}$$

meaning that in Coulomb gauge, the scalar potential is not a dynamical variable, but it is fixed to the charge density.

Let us write the solution of the electrostatic problem in a gauge-invariant form as:

10 $$\begin{aligned} {\mathbf{E }}_{\Vert }(\mathbf{x },t)=-\frac{1}{4\pi \varepsilon _0}\sum _{\alpha =0}^Zq_\alpha \frac{\left( {\mathbf{x }}_\alpha (t)-{\mathbf{x }}\right) }{| {\mathbf{x }}_\alpha (t)-{\mathbf{x }} |^3}. \end{aligned}$$

### The Lagrangian

The Lagrangian of nonrelativistic electrodynamics is usually written in the form

11 $$\begin{aligned} L({\mathbf{x }}_\alpha ,\dot{{\mathbf{x }}}_\alpha ,?) = \sum _{\alpha =0}^{Z} \frac{m_{\alpha }}{2} \, {\dot{\mathbf{x }}}_\alpha ^2 + \int \hbox {d}^{3}{x}\left[ \frac{\varepsilon _0}{2} \, {\mathbf{E }}^{2} - \frac{1}{2 \, \mu _0} \, {\mathbf{B }}^2 \right] + \int \hbox {d}^{3}{x}\, \left[ {\mathbf{j }} \cdot {\mathbf{A }} - \rho \, \Phi \right] . \end{aligned}$$

Although Maxwell’s equations and the Lorentz force can be derived *formally* by the variational method hence, the Lagrangian does not make sense per se, only as function (functional) of well-defined generalized coordinates. In this form, if the field dynamics was treated with the full potentials as coordinates then there would be a redundancy and interdependence of coordinates (cf.^10^, Section II.B.3.c). Indeed, the Maxwell equations exhibit only four dynamical field variables (the two components each of the transverse part of the two physical fields), while the a priori description by potentials gives eight (three components of the vector and one of the scalar potential plus as many components of generalized velocities).

An appropriate way to reduce the redundancy is to fix the gauge to the Coulomb gauge:

12 $$\begin{aligned} L_{\text {C}}= & {} L({\mathbf{x }}_\alpha ,\dot{{\mathbf{x }}}_{\alpha },{\mathbf{A }}_{\text {C}},\partial _{t}{\mathbf{A }}_{\text {C}}) \nonumber \\= & {} \sum _{\alpha =0}^{Z} \left( \frac{m_{\alpha }}{2} \, {\dot{\mathbf{x }}}_\alpha ^2 - \frac{1}{4\pi \varepsilon _0}\sum _{\beta =\alpha +1}^Z \frac{q_\alpha \,q_\beta }{| {\mathbf{x }}_\alpha -{\mathbf{x }}_\beta |} \right) \nonumber \\&+ \int \hbox {d}^{3}{x}\left[ \frac{\varepsilon _0}{2} \, {\mathbf{E }}_{\bot }^2 - \frac{1}{2 \, \mu _0} \, {\mathbf{B }}^2 \right] + \int \hbox {d}^{3}{x}\,{\mathbf{j }}_{\bot }\cdot {\mathbf{A }}_{\text {C}}. \end{aligned}$$

Here, to calculate the electrostatic term (second term within the summation over $\alpha$), the terms containing ${\mathbf{E }}_{\Vert }^2$ and $\rho \,\Phi$ had to be rewritten with the help of Eqs. (8b) and (9). In this form of the Lagrangian, the first term defines the atom, the second the field, and the third the interaction between these two. All these terms are gauge invariant; furthermore, the generalized field coordinate is also gauge invariant in the sense that ${\mathbf{A }}_{\text {C}}={\mathbf{A }}_{\bot }$ and ${\mathbf{A }}_{\bot }$ is gauge invariant because the transformation (6b) touches only the longitudinal part of the vector potential.

Therefore, although we invoked the Coulomb gauge as a methodological step to obtain Eq. (12) from Eq. (11), there is no need to make any consideration of gauge when working with this form of the Lagrangian. In fact, we could have solved the electrostatic part of the problem in any gauge, and arrive at the same Lagrangian. The reason why the Coulomb gauge was invoked is solely because it is in this gauge that the electrostatic problem is the easiest.

To emphasize the fact of term-by-term gauge invariance, the use of gauge-invariant coordinate, and the overall irrelevance of the choice of gauge, we reexpress the Lagrangian (12) in the manifestly gauge-invariant form:

13 $$\begin{aligned} L({\mathbf{x }}_{\alpha },\dot{\mathbf{x }}_{\alpha },{\mathbf{A }}_{\bot },\partial _{t}{\mathbf{A }}_{\bot })= & {} \sum _{\alpha =0}^{Z}\left( \frac{m_{\alpha }}{2} \, {\dot{\mathbf{x }}}_\alpha ^2 - \frac{1}{4\pi \varepsilon _0}\sum _{\beta =\alpha +1}^Z \frac{q_\alpha \,q_\beta }{| {\mathbf{x }}_\alpha -{\mathbf{x }}_\beta |} \right) \nonumber \\&+ \int \hbox {d}^{3}{x}\left[ \frac{\varepsilon _0}{2} \, {\mathbf{E }}_{\bot }^2 - \frac{1}{2 \, \mu _0} \, {\mathbf{B }}^2 \right] + \int \hbox {d}^{3}{x}\,{\mathbf{j }}_{\bot }\cdot {\mathbf{A }}_{\bot }, \end{aligned}$$

where the field coordinate ${\mathbf{A }}_{\bot }$ denotes the transverse part of the vector potential.

When considering the Lagrangian (13) as the starting point of non-relativistic (or, molecular) quantum electrodynamics in the following, then the choice of ${\mathbf{A }}_{\bot }$ as the field generalized coordinate has nothing to do with gauge fixing, because we do not say that ${\mathbf{A }}_{\Vert }$ is zero. We merely say that ${\mathbf{A }}_{\Vert }$ belongs to the electro*static* part of the problem (that is, it is not a dynamical field variable). This is true in any gauge. The Lagrangian is written after solving the electrostatic part in a gauge-invariant manner, cf. Eq. (10). When using this equation, we do not need to say anything about the expression of $\Phi$ and ${\mathbf{A }}_{\Vert }$. From Lagrangian (13), the equations of motion (1) and (4) can be derived as Euler–Lagrange equations in any gauge.

Note: It sometimes leads to misundestandings, so we emphasize that there is no general formula that expresses gauge transformation on the Lagrangian level. Indeed, if we consider the Lagrangian (11), then we see that its transformation under a gauge transformation is given as

14 $$\begin{aligned} \Delta L = - \int \hbox {d}^{3}{x}\,\left[ {\mathbf{j }} \cdot \varvec{\nabla }\chi + \rho \, \partial _t \chi \right] = \int \hbox {d}^{3}{x}\, \left[ \varvec{\nabla } \cdot {\mathbf{j }} + \partial _t \rho \right] \chi - \frac{\hbox {d}}{\hbox {d}t}\int \hbox {d}^{3}{x}\rho \, \chi = -\frac{\hbox {d}}{\hbox {d}t}\sum _{\alpha =0}^{Z} q_\alpha \chi (\mathbf{x }_\alpha (t),t) , \end{aligned}$$

where we have performed integration by parts both in space and time. After the second equality sign, the first correction term vanishes due to the continuity Eq. (3), while the second can be evaluated through Eq. (2a) to obtain the final expression. So this is a nonzero change, which is however a special case of equivalent transformations, cf. Eq. (16). On the other hand, the change of the Lagrangian (13) under gauge transformation is zero.

Note also that to obtain Eq. (13), the electrostatic part of the problem was solved in free space. However, the same program could be performed in a domain of arbitrary topology following the strategy outlined in^9^.

The Lagrangian density $\mathcal {L}$ for the field can be introduced through the definition:

15 $$\begin{aligned} L=L_\text {matter} + \int \hbox {d}^{3}{x}\, \left( \mathcal {L}_\text {field} + \mathcal {L}_\text {interaction}\right) . \end{aligned}$$

## The Power–Zienau–Woolley picture

Originally, the Power–Zienau–Woolley transformation was introduced as a unitary transformation from the minimal-coupling Hamiltonian into the so-called multipolar Hamiltonian. Since this transformation acts on the Hamiltonian and other operators expressing physical quantities, as well as the state vectors forming the Hilbert space, we refer to the resulting description as the Power–Zienau–Woolley *picture*.

In this section, starting from the gauge-invariant Lagrangian (13), we present the PZW transformation as an equivalent transformation of the Lagrangian under the form (cf. ^13^, Section I.1):

16a $$\begin{aligned} L'=L+\frac{\hbox {d}}{\hbox {d}t}\left( {\,anything\,that\,depends\,only\,on\,time\,and\,the\,generalized\,coordinates}\right) \end{aligned}$$

In the language of the action, such an equivalent transformation reads

16b $$\begin{aligned} S' = \int \limits _{t_{\text {i}}}^{t_{\text {f}}} \hbox {d}{t}L' = S + \left( {\,anything\,that\, depends\,only\,on\,t_{\text {i}}\ \,and\,t_{\text {f}}\ \,and\,the\,values\,of\,coordinates\, at\,these\,instants}\right) . \end{aligned}$$

The action $S'$ is equivalent to $S$ in the sense that it leads to the same equations of motion, due to the fact that variations of generalized coordinates and velocities vanish at the extremal time instants $t_{\text {i}}$ and $t_{\text {f}}$ according to the variational lore.

First, we introduce the polarization and magnetization fields which are key quantities in the PZW picture to express the charge and current densities.

### Polarization and magnetization fields

Let us start with the charge density. Upon introducing the polarization field as

17 $$\begin{aligned} {\mathbf{P }} (\mathbf{x },t) = \sum _{\alpha =0}^{Z} q_{\alpha } \, \varvec{\xi }_\alpha (t) \, \int \limits _{0}^{1} \hbox {d}{s}\, \delta \left( \mathbf{x }- \mathbf{x }_{\text {CoM}}- s \, \varvec{\xi }_\alpha (t) \right) , \end{aligned}$$

where $\mathbf{x }_{\text {CoM}}$ is the position of the atomic center-of-mass and $\varvec{\xi }_\alpha$s are the relative coordinates, it is found that

18 $$\begin{aligned} \rho = - \varvec{\nabla } \cdot \mathbf{P } . \end{aligned}$$

For the physical picture behind the polarization field, cf.^10^, Section IV.C.1.

In the following, we assume that the atomic nucleus is so heavy that it sits at the center of mass—which we equate with the origin—and is immobile. This is merely in order to simplify notation, e.g.:

19 $$\begin{aligned} {\mathbf{P }} (\mathbf{x },t) = \sum _{\alpha =1}^{Z} q_{\alpha } \, \mathbf{x }_\alpha (t) \, \int \limits _{0}^{1} \hbox {d}{s}\, \delta \left( \mathbf{x }- s \, \mathbf{x }_\alpha (t) \right) . \end{aligned}$$

In the next step, we play a similar game with the current density. Introducing the magnetization field as:

20 $$\begin{aligned} {\mathbf{M }} (\mathbf{x },t) = \sum _{\alpha =1}^{Z} q_{\alpha } \mathbf{x }_\alpha (t) \times {\dot{\mathbf{x }}}_\alpha (t) \, \int \limits _{0}^{1} \hbox {d}{s}\, s \, \delta \left( \mathbf{x }- s \, \mathbf{x }_\alpha (t) \right) , \end{aligned}$$

we find that

21 $$\begin{aligned} {\mathbf{j }} = \partial _t {\mathbf{P }} + \varvec{\nabla } \times {\mathbf{M }} . \end{aligned}$$

That is, the current density consists of two terms, one related to the electric polarization, the other to the magnetization of the atom.

### Power–Zienau–Woolley transformation on the Lagrangian

The electrostatic term in the Lagrangian (13) can be rewritten with the help of ${\mathbf{E }}_{\Vert }=-\varepsilon _0{\mathbf{P }}_{\Vert }$ (which follows from Eqs. (1d) and (18)) to give

22 $$\begin{aligned} \frac{1}{4\pi \varepsilon _0}\sum _{\underset{\alpha <\beta \le Z}{\alpha =0}}^Z\frac{q_\alpha \,q_\beta }{| \mathbf{x }_{\alpha }-{\mathbf{x }}_\beta |}=\int \hbox {d}^{3}{x}\frac{1}{2} \, \rho \, \Phi _{\text {C}}= - \int \hbox {d}^{3}{x}\, \frac{\varepsilon _0}{2} \, {\mathbf{E }}_{\Vert }^2 - \int \hbox {d}^{3}{x}\, {\mathbf{P }}_{\Vert }\cdot {\mathbf{E }}_{\Vert } . \end{aligned}$$

Then, using Eq. (21), we can rewrite Eq. (13) as

23 $$\begin{aligned} L= & {} \sum _{\alpha =0}^{Z} \frac{m_{\alpha }}{2} \, \dot{\mathbf{x }}_\alpha ^2 + \int \hbox {d}^{3}{x}\,\left[ \frac{\varepsilon _0}{2} \, {\mathbf{E }}^2 - \frac{1}{2 \, \mu _0} \, {\mathbf{B }}^2 \right] \nonumber \\&+ \int \hbox {d}^{3}{x}\, \left\{ \partial _{t} {\mathbf{P }}_{\bot }\cdot {\mathbf{A }}_{\bot }+ \left[ \varvec{\nabla } \times {\mathbf{M }} \right] \cdot {\mathbf{A }}\right\} + \int \hbox {d}^{3}{x}\, {\mathbf{P }}_{\Vert }\cdot {\mathbf{E }}_{\Vert } . \end{aligned}$$

Now let us perform the PZW transformation in the form of an equivalent Lagrangian transformation (16):

24 $$\begin{aligned} L_{\text {PZW}}= \,& {} L - \frac{\hbox {d}}{\hbox {d}t}\int \hbox {d}^{3}{x}\,{\mathbf{P }}_{\bot }\cdot {\mathbf{A }}_{\bot }\nonumber \\=\, & {} L - \int \hbox {d}^{3}{x}\left( \partial _{t}{\mathbf{P }}_{\bot }\right) \cdot {\mathbf{A }}_{\bot }-\int \hbox {d}^{3}{x}{\mathbf{P }}_{\bot }\cdot \left( \partial _{t}{\mathbf{A }}_{\bot }\right) . \end{aligned}$$

Performing the integration by part $\int \hbox {d}^{3}{x}\, \left[ \varvec{\nabla } \times {\mathbf{M }} \right] \cdot {\mathbf{A }}_{\bot }= \int \hbox {d}^{3}{x}\, {\mathbf{M }} \cdot \left[ \varvec{\nabla } \times \mathbf{A }_{\bot }\right]$ and substituting the physical fields in place of the vector potential, we finally obtain the new Lagrangian:

25 $$\begin{aligned} L_{\text {PZW}}= \sum _{\alpha =0}^{Z} \, \frac{m_{\alpha }}{2} \, {\dot{\mathbf{x }}}_\alpha ^2 + \int \hbox {d}^{3}{x}\, \left[ \frac{\varepsilon _0}{2} \, {\mathbf{E }}^2 - \frac{1}{2 \, \mu _0} \, {\mathbf{B }}^2 \right] + \int \hbox {d}^{3}{x}\big [ {\mathbf{P }} \cdot {\mathbf{E }} + {\mathbf{M }} \cdot {\mathbf{B }} \big ] . \end{aligned}$$

For completeness, in the following subsection we derive the familiar PZW Hamiltonian, thereby proving a posteriori that this is indeed the Lagrangian in PZW picture. However, the equivalence of the PZW picture and the gauge-invariant description with the minimal-coupling Lagrangian (13) is manifested already here on the Lagrangian level. Since the description on the Lagrangian level does not involve choices of canonical momentum, furthermore, the two Lagrangian functionals $L$ and $L_{\text {PZW}}$ together with their variables, the field coordinates are gauge invariant, there cannot be such inconsistencies in the PZW picture as alleged in Ref.^1^.

### The Hamiltonian in the PZW picture

To construct the Hamiltonian in the PZW picture, we first have to determine the canonical momenta. The Lagrangian as a function of the generalized coordinates and velocities reads:

26 $$\begin{aligned} L_{\text {PZW}}(\mathbf{x }_\alpha ,\dot{\mathbf{x }}_\alpha ,\mathbf{A }_{\bot },\partial _t\mathbf{A }_{\bot }) = \sum _{\alpha =1}^{Z} \, \frac{m_\alpha }{2} \, {\dot{\mathbf{x }}}_\alpha ^2 + \int \hbox {d}^{3}{x}\left[ \frac{\varepsilon _0}{2} \, \mathbf{E }^2 - \frac{1}{2 \, \mu _0} \, \mathbf{B }^2 \right] + \int \hbox {d}^{3}{x}\big [\mathbf{P } \cdot \mathbf{E } + \mathbf{M } \cdot \mathbf{B } \big ] , \end{aligned}$$

where we are again assuming the nucleus as immobile, this time also in the kinetic part. Here, on the left-hand-side, we again made explicit the fact that the field generalized coordinate remains $\mathbf{A }_{\bot }$ also in this picture, while the right-hand-side is written in a nice compact form with the physical fields, $\mathbf{B }$ containing the field coordinate while $\mathbf{E }$ the velocity (cf. Eq. (5)).

Canonical momenta are produced as functional derivatives of the Lagrangian along the generalized velocities:

27a $$\begin{aligned} \mathbf{p }_{\text {PZW},\alpha }&= \frac{\delta L_{\text {PZW}}}{\delta {\dot{\mathbf{x }}}_\alpha } , \end{aligned}$$

27b $$\begin{aligned} {\varvec{\Pi }}_{\text {PZW}}&= \frac{\delta L_{\text {PZW}}}{\delta \left( \partial _t \, \mathbf{A }_{\bot }\right) } . \end{aligned}$$

When evaluating the functional derivative along the particle velocity, we have to take care to differentiate also the term containing the magnetization. This term can be written in the following form:

28 $$\begin{aligned} \int \hbox {d}^{3}{x}\mathbf{M }\cdot \mathbf{B }=-\sum _{\alpha =1}^{Z}q_\alpha \,{{\dot{\mathbf{x }}}}_\alpha (t)\cdot \left[ \mathbf{x }_\alpha (t)\times \int \limits _{0}^{1} \hbox {d}{s}\, s \, \mathbf{B } \left( s \, \mathbf{x }_\alpha (t), t \right) \right] . \end{aligned}$$

In this form, the variation along the particle velocity is easily performed to yield the magnetic contribution for the particle momentum, which reads

29 $$\begin{aligned} \mathbf{p }_{\text {PZW},\alpha } (t) = m_\alpha \, {\dot{\mathbf{x }}}_\alpha (t) - q_\alpha \, \mathbf{x }_\alpha (t) \times \int \limits _{0}^{1} \hbox {d}{s}\, s \, \mathbf{B } \left( s \, \mathbf{x }_\alpha (t), t \right) . \end{aligned}$$

To calculate the field canonical momentum, let us identify the terms in $L_{\text {PZW}}$ which contain $\mathbf{E }_{\bot }=-\partial _t\,\mathbf{A }_{\bot }$:

30 $$\begin{aligned} L_{\text {PZW}}= \left( {particle\,kinetic\,terms}\right) + \left( {magnetic\, terms}\right) + \int \hbox {d}^{3}{x}\left[ \frac{\varepsilon _0}{2}\left( \mathbf{E }_{\Vert }^2+\mathbf{E }_{\bot }^2 \right) +\left( \mathbf{P }_{\Vert }\cdot \mathbf{E }_{\Vert }+\mathbf{P }_{\bot }\cdot \mathbf{E }_{\bot }\right) \right] , \end{aligned}$$

whence

31 $$\begin{aligned} {\varvec{\Pi }}_{\text {PZW}}=\frac{\delta L_{\text {PZW}}}{\delta \left( \partial _t \, \mathbf{A }_{\bot }\right) }= -\frac{\delta L_{\text {PZW}}}{\delta \mathbf{E }_{\bot }}=-\varepsilon _0\mathbf{E }_{\bot }-\mathbf{P }_{\bot }=-\mathbf{D }_{\bot }=-\mathbf{D }. \end{aligned}$$

**Figure 2:**
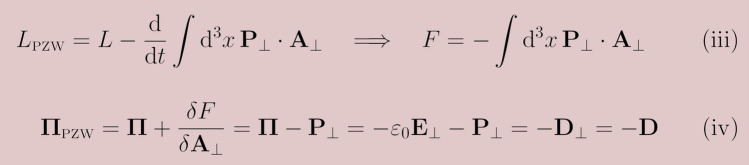
The change of conjugate momentum under the PZW transformation as derived from the general formula presented in Fig. 1.

The same result can be obtained by using the general formula of the change of momentum under an equivalent Lagrangian transformation, as we demonstrate in Fig. 2.

Having the expressions for the momenta, we can finally determine the Hamiltonian in the PZW picture:

32 $$\begin{aligned} H_{\text {PZW}}= & {} \sum _{\alpha =1}^{Z} \mathbf{p }_{\text {PZW},\alpha }(t) \cdot {\dot{\mathbf{x }}}_\alpha (t) + \int \hbox {d}^{3}{x}\, {\varvec{\Pi }}_{\text {PZW}}\cdot \partial _t \, \mathbf{A }_{\bot }\, - L_{\text {PZW}}=\sum _{\alpha =1}^{Z} \, \frac{m_\alpha }{2} \, {\dot{\mathbf{x }}}_\alpha ^2 (t) + \int \hbox {d}^{3}{x}\left( \frac{\varepsilon _0}{2}\, \mathbf{E }^2 + \frac{1}{2 \, \mu _0} \, \mathbf{B }^2 \right) \nonumber \\= & {} \sum _{\alpha =1}^{Z} \, \frac{1}{2 \, m_\alpha } \, \left( \mathbf{p }_{\text {PZW},\alpha } (t) + q_\alpha \, \mathbf{x }_\alpha (t) \times \int \limits _{0}^{1} \hbox {d}{s}\, s \, \mathbf{B } \left( s \, \mathbf{x }_\alpha (t) \right) \right) ^2 + \int \hbox {d}^{3}{x}\left( \frac{1}{2 \, \varepsilon _0} \, \left[ {\varvec{\Pi }}_{\text {PZW}}+ \mathbf{P } \right] ^2 + \frac{1}{2 \, \mu _0} \, \mathbf{B }^2 \right) \nonumber \\= & {} \sum _{\alpha =1}^{Z} \, \frac{1}{2 \, m_\alpha } \, \left( \mathbf{p }_{\text {PZW},\alpha } (t) + q_\alpha \, \mathbf{x }_\alpha (t) \times \int \limits _{0}^{1} \hbox {d}{s}\, s \, \mathbf{B } \left( s \, \mathbf{x }_\alpha (t) \right) \right) ^2\nonumber \\&+ \int \hbox {d}^{3}{x}\left( \frac{1}{2 \, \varepsilon _0} \mathbf{D }^2 + \frac{1}{2 \, \mu _0} \, \mathbf{B }^2 \right) - \frac{1}{\varepsilon _0}\int \hbox {d}^{3}{x}\mathbf{D }\cdot \mathbf{P } + \frac{1}{2\,\varepsilon _0}\int \hbox {d}^{3}{x}\mathbf{P }^2 , \end{aligned}$$

where the field canonical coordinate is contained by $\mathbf{B } = \varvec{\nabla } \times \mathbf{A }_{\bot }$. After the last equality sign, we can discover the familiar PZW Hamiltonian, which

1. is free from an electric A-square term (the magnetic contribution to the particle kinetic term is the so-called Röntgen term that vanishes in electric-dipole order),
2. accounts for the light–matter interaction in the form of the $\mathbf{D }\cdot \mathbf{P }$ term, and
3. contains a P-square term, for which a straightforward regularization procedure was presented in^14^.

## Ambiguity of the field momenta

In this section, we show that even if the field generalized coordinate is fixed (say, to $\mathbf{A }_{\bot }$), there is still a certain freedom in choosing the momentum conjugate to it, which freedom is of a field-theoretic nature. This is most easily shown in the case of the gauge-invariant Lagrangian (13). Using Eq. (8a) we can identify that part of the Largangian which contains the field velocity:

33 $$\begin{aligned} L=\int \hbox {d}^{3}{x}\frac{\varepsilon _0}{2}\,\mathbf{E }_{\bot }^2+\cdots , \end{aligned}$$

and immediately derive the field momentum conjugate to the gauge-invariant coordinate $\mathbf{A }_{\bot }$ to obtain

34 $$\begin{aligned} {\varvec{\Pi }}=\frac{\delta \mathcal {L}_\text {field}}{\delta \left( \partial _t \mathbf{A }_{\bot }\right) }=-\varepsilon _0\,\mathbf{E }_{\bot }. \end{aligned}$$

However, this part of the Lagrangian could be supplemented as

35 $$\begin{aligned} L=\int \hbox {d}^{3}{x}\left( \frac{\varepsilon _0}{2}\,\mathbf{E }_{\bot }^2+\varepsilon _0\,\mathbf{E }_{\Vert }\cdot \mathbf{E }_{\bot }\right) +\cdots , \end{aligned}$$

since the spatial integral of the second term is zero. Varying this form, we find

36 $$\begin{aligned} \delta L= & {} \int \hbox {d}^{3}{x}\left( \varepsilon _0\,\mathbf{E }_{\bot }\cdot \delta \mathbf{E }_{\bot }+\varepsilon _0\,\mathbf{E }_{\Vert }\cdot \delta \mathbf{E }_{\bot }\right) =-\int \hbox {d}^{3}{x}\varepsilon _0\left( \mathbf{E }_{\bot }+\mathbf{E }_{\Vert }\right) \cdot \delta \left( \partial _t\mathbf{A }_{\bot }\right) \nonumber \\= & {} -\int \hbox {d}^{3}{x}\varepsilon _0\,\mathbf{E }\cdot \delta \left( \partial _t\mathbf{A }_{\bot }\right) , \end{aligned}$$

yielding ${\varvec{\Pi }}=-\varepsilon _0\,\mathbf{E }$ for the canonical field momentum. This is also the a priori result from the generic Lagrangian Eq. (11), when the variation is performed symbolically without regard to the interdependence of the variables. This is also one of the starting points of the paper by Rousseau and Felbacq^1^. However, here we clarified that this is only one of several possible choices.

As explained by Weinberg in Section 11.3 of his book^15^, the choice of ${\varvec{\Pi }}'=-\varepsilon _0\,\mathbf{E }$ as canonical field momentum has the “awkward feature” (quote: Weinberg) that when quantized, it does not commute with the particle momenta, for the simple reason that $\mathbf{E }_{\Vert }$ is determined by the particle coordinates. On the other hand, with the choice ${\varvec{\Pi }}=-\varepsilon _0\,\mathbf{E }_{\bot }$, the only nontrivial commutation relation will be the well-known (cf. Eq. (11.3.20) in^15^)

37 $$\begin{aligned} \left[ \mathbf{A }_{\bot }(\mathbf{x },t) , {\varvec{\Pi }}(\mathbf{y },t)\right] =i\hbar \, \varvec{\delta }_{\bot }\left( \mathbf{x }-\mathbf{y }\right) . \end{aligned}$$

Here, $\varvec{\delta }_{\bot }$ is the transverse delta function, a 2nd-order tensor field.

Let us note that what we are doing here with Eq. (35) is different from equivalent transformations of the Lagrangian (cf. Eq. (16)), since here the change of the Lagrangian under the switch between the two possible choices of field momentum is effectively zero, so that for example the picture cannot change either.

In the following, we show that a similar freedom is present in the PZW picture.

### The connection with Poincaré gauge

The defining condition of Poincaré gauge reads:

38 $$\begin{aligned} \mathbf{x }\cdot \mathbf{A }_{\text {P}}=0 . \end{aligned}$$

This condition is equivalent to a pair of expressions whereby the potentials are uniquely determined by the physical fields in this gauge:

39a $$\begin{aligned} \Phi _{\text {P}}&= \Phi _0 (t) - \mathbf{x }\cdot \int \limits _{0}^{1} \hbox {d}{s}\, \mathbf{E } \left( s \, \mathbf{x },t \right) , \end{aligned}$$

39b $$\begin{aligned} \mathbf{A }_{\text {P}}&= - \mathbf{x }\times \int \limits _{0}^{1} \hbox {d}{s}\, s \, \mathbf{B } \left( s \, \mathbf{x },t \right) , \end{aligned}$$

where $\Phi _0 (t)$ is an integration constant which, being independent of space, does not enter the physical fields.

Note that from Eq. (39b), the transverse vector potential determines the whole of $\mathbf{A }_{\text {P}}$ by virtue of $\mathbf{B }=\varvec{\nabla } \times \mathbf{A }=\varvec{\nabla } \times \mathbf{A }_{\bot }$, which underlines that $\mathbf{A }_{\bot }$ is the true dynamical variable also in Poincaré gauge. We also note that the physical fields always uniquely determine the potentials with different expressions in any gauge.

It is clear that Eq. (38) follows from Eq. (39b), while the other direction of the equivalence is proven in Sec. A of the Supplementary Material.

Let us return to the “generic” formula of the Lagrangian (11), and substitute the expressions (39). The interaction terms then read:

40a $$\begin{aligned} \int \hbox {d}^{3}{x}\, \rho \, \Phi _{\text {P}}=&- \sum _{\alpha =1}^{Z} \, q_{\alpha } \,\mathbf{x }_\alpha (t) \cdot \int \limits _{0}^{1} \hbox {d}{s}\, \mathbf{E }\left( s \, \mathbf{x }_\alpha (t),t\right)= & {} - \int \hbox {d}^{3}{x}\, \mathbf{P } \cdot \mathbf{E } , \end{aligned}$$

40b $$\begin{aligned} \int \hbox {d}^{3}{x}\, \mathbf{j } \cdot \mathbf{A }_{\text {P}}=&- \sum _{\alpha =1}^{Z} \, q_{\alpha } {\dot{\mathbf{x }}}_\alpha (t) \cdot \left[ \mathbf{x }_\alpha (t) \times \int \limits _{0}^{1} \hbox {d}{s}\, s \, \mathbf{B } \left( s \, \mathbf{x }_\alpha (t),t \right) \right]= & {} \int \hbox {d}^{3}{x}\, \mathbf{M } \cdot \mathbf{B } , \end{aligned}$$

where we have used that the term containing $\Phi _0 (t)$ vanishes due to the neutrality of the atom, while the second equality in each row comes from the comparison with Eqs. (19) and (20). This means that by expressing the interaction terms with the potentials in Poincaré gauge, we symbolically get the form Eq. (25) of the Lagrangian:

41 $$\begin{aligned} L_{\text {Poincar}\acute{e}}(\mathbf{x }_\alpha ,\dot{\mathbf{x }}_\alpha ,?)\overset{\text {symbolically}}{=}L_{\text {PZW}}(\mathbf{x }_\alpha ,\dot{\mathbf{x }}_\alpha ,\mathbf{A }_{\bot },\partial _t\mathbf{A }_{\bot }). \end{aligned}$$

What is important to note here is that though we have fixed the gauge in the Lagrangian (11) in the sense that we substituted the forms of the potentials in a certain gauge, we haven’t declared yet with what generalized coordinates we intend to describe the dynamics of the field. Gauge fixing and the choice of coordinates are two conceptually different things: the first fixes the expressions of the potentials, however, in the Lagrangian description of electrodynamics, nothing obliges us to use the (full) potentials as field coordinates!

The transverse part of the vector potential remains a good choice for the field coordinate also in Poincaré gauge. It is gauge invariant, i.e. ${\mathbf{A }}_{\text {P}}{,_{\bot }}={\mathbf{A }}_{\bot }(={\mathbf{A }}_{\text {C}}{,_{\bot }}(={\mathbf{A }}_{\text {C}}))$, but, more importantly, it bijectively covers the c-manifold of the field. With this choice it is possible to say also in the physical sense that

42 $$\begin{aligned} L_{\text {Poincar}\acute{e}}({\mathbf{x }}_{\alpha },\dot{\mathbf{x }}_{\alpha },{\mathbf{A }}_{\bot },\partial _t{\mathbf{A }}_{\bot })=L_{\text {PZW}}({\mathbf{x }}_{\alpha },\dot{\mathbf{x }}_{\alpha },{\mathbf{A }}_{\bot },\partial _{t}{\mathbf{A }}_{\bot })\quad \text {for a single atom}. \end{aligned}$$

We see that even in this case, the equivalence of the Poincaré-gauge and the PZW Lagrangian comes with a qualification.

Note that the PZW picture is easily extended to the case of several atoms = several charge centres, e.g. the polarization field can be chosen as

43 $$\begin{aligned} \mathbf{P } = \sum _A\sum _{\alpha \in A} q_{\alpha } \,\varvec{\xi }_{A,\alpha } (t) \, \int \limits _{0}^{1} \hbox {d}{s}\, \delta \left( \mathbf{x }- \mathbf{x }_{\text {CoM},A} - s \, \varvec{\xi }_{A,\alpha } (t) \right) , \end{aligned}$$

where $A$ indexes the different atoms—this form with separate charge centres for the different atoms being a convenient starting point for a multipolar approximation. But the Poincaré gauge defined by

44 $$\begin{aligned} \left( \mathbf{x }-\mathbf{x }_{\text {CoM}}\right) \cdot \mathbf{A }_{\text {P}}=0 \end{aligned}$$

knows about a single centre of charge only. This only expresses the fact well-known in literature (cf. e.g.^10^ Chapter IV.) that the set of equivalent transformations of the Lagrangian is wider than that of gauge transformations.

Let us now turn to the conjugate momentum in Poincaré gauge, where we can show that a similar ambiguity is present as noted above in the case of the gauge-invariant Lagrangian. As we have shown in Eq. (31), the natural choice in Poincaré gauge is ${\varvec{\Pi }}_{\text {P}}=-\mathbf{D }$. However, using the identity

45 $$\begin{aligned} \int \hbox {d}^{3}{x}\mathbf{P }_{\bot }\cdot \mathbf{E }_{\bot }=\int \hbox {d}^{3}{x}\mathbf{P }_{\Vert }\cdot \left( \partial _t\mathbf{A }_{\text {P}}\right) _{\Vert }, \end{aligned}$$

whose proof is given in Sec. B of the Supplementary Material, in the Lagrangian (30) one of the appearances of the field generalized velocity can be eliminated to give

46 $$\begin{aligned} L_{\text {P}}= \left( {\,particle\,kinetic\,terms}\right) + \left( {\,magnetic\,terms}\right) + \int \hbox {d}^{3}{x}\left[ \frac{\varepsilon _0}{2}\left( \mathbf{E }_{\Vert }^2+\mathbf{E }_{\bot }^2\right) +\mathbf{P }_{\Vert }\cdot \left( \mathbf{E }_{\Vert }+\left( \partial _t\mathbf{A }_{\text {P}}\right) _{\Vert }\right) \right] . \end{aligned}$$

Now the subscript $\text {P}$ refers to either Poincaré gauge or PZW picture in the just discussed sense of equivalence between the two. What we have to note here is that $\mathbf{A }_{\text {P}}{,_{\Vert }}$ is not a field variable but one that belongs to the electrostatic part of the problem. This can be immediately seen from Eq. (5a) because $\mathbf{E }_{\bot }$ is determined solely by $\mathbf{A }_{\bot }$ also in Poincaré gauge (both of these vector fields are in fact gauge invariant), while $\Phi _{\text {P}}$ and $\mathbf{A }_{\text {P}}{,_{\Vert }}$ conspire to yield $\mathbf{E }_{\Vert }$, which latter is determined by the particles (here $\mathbf{E }_{\Vert }$ is gauge invariant, but $\Phi$ and $\mathbf{A }_{\Vert }$ are of course not). The derivation (31) is modified when we start out from the form (46) to yield ${\varvec{\Pi }}_{\text {P}}'=-\varepsilon _0\,\mathbf{E }_{\bot }$ for the canonical field momentum.

How do we decide which momentum variable to use in this case? Here we cannot rely on the argument of convenience as in the case of the minimal-coupling picture above, since here the commutation relations will be the same with both choices:

47 $$\begin{aligned} \left[ \mathbf{A }_{\bot }(\mathbf{x },t) , -\mathbf{D }(\mathbf{y },t)\right]= \,& {} \left[ \mathbf{A }_{\bot }(\mathbf{x },t) , {\varvec{\Pi }} _{\text {P}}(\mathbf{y },t)\right] \nonumber \\=\, & {} i\hbar \,\varvec{\delta }_{\bot }\left( \mathbf{x }-\mathbf{y }\right) \nonumber \\= \,& {} \left[ \mathbf{A }_{\bot }(\mathbf{x },t) , {\varvec{\Pi }}_{\text {P}}'(\mathbf{y },t)\right] =\left[ \mathbf{A }_{\bot }(\mathbf{x },t) , -\varepsilon _0\mathbf{E }_{\bot }(\mathbf{y },t)\right] . \end{aligned}$$

The answer is that if we are aiming at the usual form of the PZW Hamiltonian (32), then we have to choose $-\mathbf{D }$ as the field canonical momentum because even though the equations of motion are independent, the *form* of the Hamiltonian does depend on the choice of momentum.

When the full potentials $\Phi _{\text {P}}$ and $\mathbf{A }_{\text {P}}$ are chosen as field coordinate, the c-manifold is not bijectively covered since $\Phi _{\text {P}}$ and part of $\mathbf{A }_{\text {P}}$ belong to the electrostatic part of the problem, which is at the same time determined also by the particle coordinates.

Note that in this case, the coordinate space spanned by the $\mathbf{x }_\alpha$s, $\Phi _{\text {P}}$, and $\mathbf{A }_{\text {P}}$ is “bigger” than the c-manifold because part of this coordinate space—where the particle coordinates, the scalar potential, and the longitudinal part of the vector potential determine the electrostatic part differently—is actually non-physical. This situation can be handled by the explicit use of the following constraint derived from Eq. (10):

48 $$\begin{aligned} \partial _t\mathbf{A }_{\text {P}}{,_{\Vert }}+\varvec{\nabla }\Phi _{\text {P}}=\frac{1}{4\pi \varepsilon _0}\sum _{\alpha =0}^Zq_\alpha \frac{ \left( \mathbf{x }_\alpha (t)-\mathbf{x }\right) }{| \mathbf{x }_\alpha (t)-\mathbf{x } |^3}. \end{aligned}$$

In this case the equivalence of the PZW picture with Poincaré gauge does not hold even in the relative sense described above:

49 $$\begin{aligned} L_{\text {Poincar}\acute{e}}(\mathbf{x }_\alpha ,\dot{\mathbf{x }}_\alpha ,\Phi _{\text {P}},\mathbf{A }_{\text {P}},\partial _t\mathbf{A }_{\text {P}})\ne L_{\text {PZW}}(\mathbf{x }_\alpha ,\dot{\mathbf{x }}_\alpha ,\mathbf{A }_{\bot },\partial _t\mathbf{A }_{\bot }), \end{aligned}$$

which can also be immediately seen because the domains of these two functionals are different.

## Summary

In summary, we have shown that the Power–Zienau–Woolley picture can be derived from a gauge-invariant Lagrangian, in a way which does not make reference to any gauge or choice of canonical momentum. For a treatment emphasizing the unitary equivalence between the minimal-coupling and the PZW picture, cf. Ref.^16^.

We believe that our analysis has clearly dissolved all the objections raised recently against the PZW picture by Rousseau and Felbacq^1^. In the following, we briefly react to some central claims of theirs which appear erroneous in the light of our treatment.

1. Talking about a “multipolar gauge” is strictly speaking not correct, and this is not how the PZW picture was understood in the literature, either^16^. As we have seen in “The connection with Poincaré gauge”, it is only in a strongly qualified sense that we can talk about the equivalence of the Poincaré gauge and the PZW picture (e.g. only in the case of a single charge centre, that is, a single atom), but such an idea can only occur if the transverse vector potential is used as field coordinate irrespective of gauge, as is the case in the PZW picture.
2. The PZW picture cannot be declared inconsistent on the basis that it is not derived via a gauge transformation. Here we have shown that the minimal-coupling picture can be formulated in a gauge-invariant manner, and the PZW picture is equivalent to this in the sense that it can be derived through an equivalent Lagrangian transformation regardless of gauge. It is furthermore a well-known fact that such transformations are more general than gauge transformations. However, the field coordinate—which is the gauge invariant $\mathbf{A }_{\bot }$ in both pictures—remains untouched by a Lagrangian transformation, as this is part of the definition of the Lagrangian problem in the first place. Moreover, both the Lagrangian (25) and the Hamiltonian (32) can be expressed solely with the physical fields in the PZW picture. When the PZW Hamiltonian is used in Schrödinger picture, as is often the case in quantum optics e.g. in the derivation of the Jaynes–Cummings model, then potentials do not play a role at all, further emphasizing the irrelevance of gauge.
3. It is incorrect to expect that the canonical momentum is gauge invariant, since the momentum in general does change under an equivalent Lagrangian transformation in the form of Eq. (16) as demonstrated in Figs. 1 and 2. Sure, the electric field $\mathbf{E }$ is gauge invariant, *but its capacity of being the canonical field momentum is not*.
4. The appearance of the displacement field $\mathbf{D }$ in the PZW picture does not mean that concepts from macroscopic electrodynamics are mixed into the electrodynamics of atoms. The replacement of the charge and current densities with polarization and magnetization densities is just another way of describing the same things.
5. It is incorrectly argued that the A-square term is present in the PZW picture. It is true that in the particle momentum (29), the magnetic contribution is exactly $\mathbf{A }_{\text {P}}$, but this is still a magnetic term, which will be neglected in electric-dipole order, so that it does not cause the same problems as the “electric” A-square term in Coulomb gauge.
  In Ref.^1^, remarkable effort is taken to calculate the Dirac brackets in the case when the full $\mathbf{A }_{\text {P}}$ is taken as field coordinate and $-\varepsilon _0\mathbf{E }$ as conjugate momentum. This is, however, an unnecessary complication, in analogy with the choice of $\mathbf{A }_{\bot }$ and the full $-\varepsilon _0\mathbf{E }$ in the minimal-coupling picture, as discussed in “Ambiguity of the field momenta”. In our treatment, in the PZW picture just like in the minimal-coupling one the only non-trivial Dirac bracket is the one between the field coordinate and conjugate momentum, and it is proportional to $\varvec{\delta }_{\bot }$ (cf. Eq. (47)).

## Supplementary Information

Supplementary Information.

## Data Availability

No datasets were generated or analysed during the current study.
