# Supplementary material for: The gauge-invariant Lagrangian, the Power–Zienau–Woolley picture, and the choices of field momenta in nonrelativistic quantum electrodynamics
Source: Sci Rep. 2021 Aug 11;11:16337. doi: 10.1038/s41598-021-94405-z (PMC8358002; doi:10.1038/s41598-021-94405-z)
Supplement: Supplementary file 1 — Supplementary Information. [file 41598_2021_94405_MOESM1_ESM.pdf]

Supplementary Material to "The gauge-invariant Lagrangian, the Power-Zienau-Woolley picture, and the choices of field momenta in nonrelativistic quantum electrodynamics"

A. Vukics,* G. Kónya, and P. Domokos

Wigner Research Centre for Physics, H-1525 Budapest, P.O. Box 49., Hungary

(Dated: December 10, 2019)

* vukics.andras@wigner.mta.hu

A: POTENTIALS FROM THE CONDITION DEFINING THE POINCARÉ GAUGE

For the definition of the Poincaré gauge, we must first choose a point of reference, which we denote by $\mathbf{x}_0$. At the point of reference, the value of the scalar potential at any time instant can be freely prescribed, let this be $\Phi_0(t)$. The condition defining the Poincaré gauge reads

$$(\mathbf{x}-\mathbf{x}_0)\cdot\mathbf{A}_\mathrm{P}(\mathbf{x},t)=0\ . \tag{1}$$

What we are proving in this Appendix is that the gauge fixing condition and the prescribed value of the scalar potential completely determine the potentials.

These two conditions can be automatically satisfied by looking for the potentials in the following form:

$$\Phi_\mathrm{P}(\mathbf{x},t)=\Phi_0(t)-(\mathbf{x}-\mathbf{x}_0)\cdot\mathbf{u}(\mathbf{x},t)\ , \tag{2a}$$

$$\mathbf{A}_\mathrm{P}(\mathbf{x},t)=-(\mathbf{x}-\mathbf{x}_0)\times\mathbf{v}(\mathbf{x},t)\ , \tag{2b}$$

where $\mathbf{u}(\mathbf{x},t)$ and $\mathbf{v}(\mathbf{x},t)$ are auxiliary vector fields. The potentials do not uniquely determine the auxiliary vector fields, as the latter can be transformed in the following way without changing the potentials:

$$\mathbf{u}'(\mathbf{x},t)=\mathbf{u}(\mathbf{x},t)+(\mathbf{x}-\mathbf{x}_0)\times\mathbf{w}(\mathbf{x},t)\ , \tag{3a}$$

$$\mathbf{v}'(\mathbf{x},t)=\mathbf{v}(\mathbf{x},t)+(\mathbf{x}-\mathbf{x}_0)\cdot\varphi(\mathbf{x},t)\ . \tag{3b}$$

In the following, this freedom in the auxiliary vector fields will be eliminated by prescribing extra conditions. Since we strive to relate $\mathbf{u}$ with $\mathbf{E}$ and $\mathbf{v}$ with $\mathbf{B}$, these extra conditions are fashioned after Maxwell's homogeneous equations as

$$\boldsymbol{\nabla}\times\mathbf{u}=-\partial_t\,\mathbf{v}\ , \tag{4a}$$

$$\boldsymbol{\nabla}\cdot\mathbf{v}=0\ . \tag{4b}$$

In the next step, let us see what equations we get for $\mathbf{u}$ and $\mathbf{v}$ if the formulas eq. (2) are substituted into Eq. (5) of the main text. To simplify notation, we choose the origin as point of reference: $\mathbf{x}_0=\mathbf{0}$. Then:

$$\mathbf{E}=\mathbf{x}\times\partial_t\mathbf{v}+\boldsymbol{\nabla}(\mathbf{x}\cdot\mathbf{u})\ , \tag{5a}$$

$$\mathbf{B}=-\boldsymbol{\nabla}\times(\mathbf{x}\times\mathbf{v})\ . \tag{5b}$$

Using eq. (4a) and expanding the vector triple products, we get after simplification (using also eq. (4b) in the expression of $\mathbf{B}$):

$$\mathbf{E}(\mathbf{x},t) = \big(\mathbf{x}\cdot\boldsymbol{\nabla}\big)\,\mathbf{u}(\mathbf{x},t) + \mathbf{u}(\mathbf{x},t)\ , \tag{6a}$$

$$\mathbf{B}(\mathbf{x},t) = \big(\mathbf{x}\cdot\boldsymbol{\nabla}\big)\,\mathbf{v}(\mathbf{x},t) + 2\,\mathbf{v}(\mathbf{x},t)\ . \tag{6b}$$

So, these two equations are to be solved for the auxiliary vector fields.

Let us perform the substitution $\mathbf{x} \to s\,\mathbf{x}$, where s is a scalar parameter:

$$\mathbf{E}(s\,\mathbf{x},t) = s\,\big(\mathbf{x}\cdot\boldsymbol{\nabla}\big)\,\mathbf{u}(s\,\mathbf{x},t) + \mathbf{u}(s\,\mathbf{x},t)\ , \tag{7a}$$

$$\mathbf{B}(s\,\mathbf{x},t) = s\,\big(\mathbf{x}\cdot\boldsymbol{\nabla}\big)\,\mathbf{v}(s\,\mathbf{x},t) + 2\,\mathbf{v}(s\,\mathbf{x},t)\ . \tag{7b}$$

The introduction of this parameter is useful as we can notice that

$$\frac{\mathrm{d}}{\mathrm{d}s}\,\mathbf{u}(s\,\mathbf{x},t) = \big(\mathbf{x}\cdot\boldsymbol{\nabla}\big)\,\mathbf{u}(s\,\mathbf{x},t)\ , \quad \text{the same being true for } \mathbf{v}, \tag{8}$$

so that the equations become ordinary differential equations along s. Let us multiply eq. (7b) with s and contract the terms on the right hand side:

$$\mathbf{E}(s\,\mathbf{x},t) = \frac{\mathrm{d}}{\mathrm{d}s}\Big(s\,\mathbf{u}(s\,\mathbf{x},t)\Big)\ , \tag{9a}$$

$$s\,\mathbf{B}(s\,\mathbf{x},t) = \frac{\mathrm{d}}{\mathrm{d}s}\Big(s^2\,\mathbf{v}(s\,\mathbf{x},t)\Big)\ . \tag{9b}$$

Let us integrate these two equations along s from 0 to 1:

$$\int_0^1 \mathrm{d}s\,\mathbf{E}(s\,\mathbf{x},t) = s\,\mathbf{u}(s\,\mathbf{x},t)\Big|_{s=0}^{1} = \mathbf{u}(\mathbf{x},t)\ , \tag{10a}$$

$$\int_0^1 \mathrm{d}s\,s\,\mathbf{B}(s\,\mathbf{x},t) = s^2\,\mathbf{v}(s\,\mathbf{x},t)\Big|_{s=0}^{1} = \mathbf{v}(\mathbf{x},t)\ . \tag{10b}$$

Whereby we have determined the two auxiliary vector fields and hence the potentials in Poincaré gauge. The only remaining task is to verify that the solutions eq. (10) satisfy the conditions eq. (4), as this is obviously required for the consistency of our solution. But the satisfaction of these extra conditions follows directly from the homogeneous Maxwell equations.

B: PROOF OF THE IDENTITY (45) OF THE MAIN TEXT

Using the expressions (19) and (39b) of the main text, we can derive

$$\int \mathrm{d}^3x\,\mathbf{P}\cdot(\partial_t\mathbf{A}_{\mathrm{P}}) = \int \mathrm{d}^3x \left[\sum_{\alpha=1}^{Z} q_\alpha\,\mathbf{x}_\alpha(t)\int_0^1 \mathrm{d}s\;\delta(\mathbf{x}-s\,\mathbf{x}_\alpha(t))\right]\cdot\left[-\mathbf{x}\times\int_0^1 \mathrm{d}s'\;s'\left(\partial_t\mathbf{B}\big(s'\,\mathbf{x}\,,t\big)\right)\right]$$
$$= -\sum_{\alpha=1}^{Z} q_\alpha\int_0^1 \mathrm{d}s\int_0^1 \mathrm{d}s'\;s'\,\mathbf{x}_\alpha(t)\cdot\left[\int \mathrm{d}^3x\;\delta(\mathbf{x}-s\,\mathbf{x}_\alpha(t))\,\mathbf{x}\times\left(\partial_t\mathbf{B}\big(s'\,\mathbf{x}\,,t\big)\right)\right]$$
$$= -\sum_{\alpha=1}^{Z} q_\alpha\int_0^1 \mathrm{d}s\,s\int_0^1 \mathrm{d}s'\;s'\,\mathbf{x}_\alpha(t)\cdot\left[\mathbf{x}_\alpha(t)\times\left(\partial_t\mathbf{B}\big(s\,s'\,\mathbf{x}_\alpha(t)\,,t\big)\right)\right], \quad (11)$$

where the scalar triple product vanishes, due to the equality of two of its vectors. Then, using the gauge invariance of the transverse part of the vector potential, we have

$$0 = \int \mathrm{d}^3x\,\mathbf{P}\cdot(\partial_t\mathbf{A}_{\mathrm{P}}) = \int \mathrm{d}^3x\,\mathbf{P}_\parallel\cdot(\partial_t\mathbf{A}_{\mathrm{P}})_\parallel + \int \mathrm{d}^3x\,\mathbf{P}_\perp\cdot(\partial_t\mathbf{A}_{\mathrm{P}})_\perp$$
$$= \int \mathrm{d}^3x\,\mathbf{P}_\parallel\cdot(\partial_t\mathbf{A}_{\mathrm{P}})_\parallel - \int \mathrm{d}^3x\,\mathbf{P}_\perp\cdot\mathbf{E}_\perp, \quad (12)$$

which is just Eq. (45) of the main text.
